# Magnetic and Biomedical Properties of Iron Nanoparticles Synthesized Using *Vitex agnus-castus* Extract

**DOI:** 10.3390/ma17246064

**Published:** 2024-12-11

**Authors:** Kadriye Kızılbey, Elif Nur Köprülü, Hatice Temür, Sezen Canım Ateş, Sevil Özer

**Affiliations:** 1 Department of Natural Sciences, Faculty of Engineering and Natural Sciences, Acıbadem University, Istanbul 34752, Türkiye; 2 Division of Biomedical Engineering, Institute of Graduate Studies, Istanbul University-Cerrahpasa, Istanbul 34320, Türkiye; elifkprlnur@gmail.com; 3 Biomedical Engineering Program, Department of Electronics and Communication Engineering, Institute of Graduate Studies, Istanbul Technical University, Istanbul 34469, Türkiye; 4 Biomedical Engineering Department, Faculty of Engineering and Architecture, Istanbul Yeni Yüzyıl University, Istanbul 34010, Türkiye

**Keywords:** biocompatibility, green synthesis, magnetic nanoparticles, cytotoxicity, anticancer activities

## Abstract

Magnetic nanoparticles have attracted significant attention in nanoscience and nanotechnology due to their unique physicochemical properties. These properties enable their great potential in various biomedical applications, such as hyperthermia, drug delivery, tissue engineering, theranostics, and lab-on-a-chip technologies. Physical and chemical methods are conventionally used for the synthesis of nanoparticles; however, due to several limitations of these methods, research focus has recently shifted towards developing clean and eco-friendly synthesis protocols while maintaining their desirable chemical and physical properties. In this study, iron oxide nanoparticles (FeNPs) were synthesized for the first time using the green synthesis method with extracts from *Vitex agnus-castus*. The structural and magnetic characterization of FeNPs was carried out using state-of-the-art techniques. The formation of FeNPs was confirmed by UV–vis spectroscopy. The morphology and size distribution were examined by a zetasizer and SEM, which showed agglomerated ring-shaped structures with a moderate size distribution among the nanoparticles. The crystalline structure and phase purity of the FeNPs were analyzed by XRD. FT-IR spectroscopy confirmed the attachment of bioactive plant molecules on the FeNP surfaces. The TGA results indicated the presence of organic molecules on the surface of the nanoparticles. Further studies including temperature-dependent magnetization and coercivity measurements were performed by PPMS and ESR, confirming the soft magnetic characteristics of synthesized FeNPs. Additionally, the dose-dependent toxicity and anti-cancerogenic effects of the FeNPs were screened towards the glioma cancer line (C6) and fibroblast cell line (L929) in vitro using an MTT assay. After 24 h of treatment, inhibitory concentration IC50 values of 26.51 µg/mL (l929) and 10.73 µg/mL (C6) were determined, respectively. These results suggest the potential of the synthesized FeNPs in developing new biocompatible systems for diagnostic and therapeutic purposes. This study contributes to the growing demand for research in nanotechnology by offering a sustainable and effective green synthesis method for FeNPs, expanding their potential applications in nanomedicine.

## 1. Introduction

The desire to produce and use tools with nanometer dimensions owing to their physical and chemical properties is increasing in a wide range of applications in various fields of biology, drugs, and medicine. Presently, researchers focus on magnetic nanoparticles because of their large surface area, low melting point, and exceptional optical, electrical, and thermal properties. In recent years, the successful application of metallic nanoparticles in cancer diagnosis and treatment has dramatically expanded [1,2]. Various physical and chemical approaches are used to synthesize metallic nanoparticles, like sol–gel, co-precipitation, hydrothermal methods, steam condensation, and micro-emulsion [3,4,5,6,7]. A promising method for the biosynthesis of iron oxide magnetic nanoparticles involves utilizing natural products, such as plant extracts, to reduce metal ions. This approach is highly scalable, cost-effective, and avoids chemical contaminants, making it ideal for medical and biological applications where nanoparticle purity is crucial [1,8]. Plants are rich in phytochemical compounds, including phenolics, terpenoids, polysaccharides, and flavonoids, which possess oxidation–reduction properties. As a result, they are widely preferred for the green synthesis of nanoparticles [9]. The use of plant extracts for nanoparticle synthesis not only increases yield, but also leverages the abundance of phytochemical components in these extracts as reducing and stabilizing agents, facilitating the conversion of metal ions into metal nanoparticles [10]. The synthesis of nanoparticles using plant extracts generally follows a standardized protocol comprising several steps. Initially, the plant material is boiled to produce an aqueous extract, with emphasis on thorough filtration to ensure a clear extract, which significantly improves the efficiency of nanoparticle synthesis. Next, a metal salt solution is combined with the aqueous extract, and the mixture is incubated. Various plant parts, including roots, stems, bark, leaves, flowers, fruits, and seeds, have been employed for the green synthesis of nanoparticles [11,12]. Among the metals commonly studied in biomaterials, gold and silver dominate, with numerous reports documenting the synthesis of these nanoparticles using extracts from diverse plant sources [2,13,14]. In contrast, the synthesis of iron nanoparticles via similar methods remains a challenge, as reduced iron is highly susceptible to oxidation in solution, making it more difficult to achieve stable particles compared to gold or silver. The present study’s novelty lies in the green synthesis and comprehensive characterization of iron nanoparticles using *Vitex agnus-castus* extract as a bio-reductant and stabilizing agent.

Vitex agnus-castus is a deciduous shrub from the Verbenaceae family [15]. It is native to Mediterranean countries and has spread to Central Asia and Southern Europe. This plant is recognized for its medicinal properties, with its fruits traditionally used in countries like Iran, Italy, Greece, and Egypt to treat various female health issues, including spasmodic dysmenorrhea and menstrual pain. Much research is endorsing the efficacy of using *Vitex agnus-castus* and reporting its analgesic, anti-inflammatory, antimicrobial, diuretic, and digestive properties [3,16,17]. In addition, antifungal activity was higher than antibacterial activity in these studies [18]. An in vitro study investigating the antioxidant and anticancer activities of diethyl ether, petroleum ether, ethyl acetate, methanol, and water (infusion and decoction) extracts from *Vitex agnus-castus* seeds revealed that the cytotoxic effects on MCF-7 breast cancer cells, DNA damage, and apoptotic activity intensified with increasing extract concentration [19]. *Vitex agnus-castus* L. is rich in bioactive metabolites, including flavonoids, ketosteroids, iridoids, and essential oils, which exhibit strong reducing properties and play a crucial role in the green synthesis of metallic nanoparticles [20]. In this study, a facile and green method to synthesize FeNPs with *Vitex agnus-castus* seed extract as the reductant was achieved for the first time. The effect of the green synthesis on the structural and magnetic properties of the resultant product was investigated in detail using Fourier-transform infrared spectroscopy (FTIR), a zetasizer, scanning electron microscopy (SEM), UV-Vis spectrometry analysis, a physical properties measurement system (PPMS), electron spin resonance spectroscopy (ESR), and thermogravimetric (TGA) and X-ray diffraction (XRD) analysis. An MTT assay was used to determine the cytotoxic potential and anticarcinogenic activity of *Vitex agnus-castus* at different concentrations.

## 2. Materials and Methods

The seeds of *Vitex agnus-castus* were obtained from a local herbalist in Turkey. They were pulverized using a grinder. Iron(III) chloride hexahydrate (FeCl_3_·6H_2_O) and sodium hydroxide (NaOH) were purchased from Sigma Aldrich, St. Louis, MI, USA.

### 2.1. Extraction of Vitex agnus-castus Plant

In total, 400 g of *Vitex agnus-castus* plant seed powder and 1000 mL of distilled water were used to prepare the extract, which was brewed at 700 rpm for 30 min at 80 °C in a heater. The extract was filtered and clarified using Wattman No. 1 filter paper.

### 2.2. Green Synthesis of FeNPs

A 0.01 M iron chloride solution was prepared by dissolving 0.15 g of FeCl_3_·6H_2_O in 80 mL of deionized water, and the mixture was stirred on a magnetic stirrer until a homogeneous solution was obtained. The resulting FeCl_3_ solution was slowly added to the 400 mL *Vitex agnus-castus* plant extract using a Pasteur pipette. Then, 45 mL of distilled water solution and 0.1 M of NaOH were prepared to adjust the mixture to a basic pH level. This solution was gradually added to the plant extract under the control of a pH meter. The mixture was centrifuged at 10,000 rpm for 10–15 min, and the supernatant was separated. The nanoparticles were washed three times with distilled water and ethanol to remove water-soluble impurities, followed by centrifugation and drying in an oven at 70 °C. After drying, the material was ground into powder form, yielding 0.81 g of FeNP powder.

### 2.3. Characterization of FeNPs

UV-Vis spectrophotometry was used to determine the optical properties of the green-synthesized FeNPs. The plant extracts and nanoparticles were analyzed at 200–800 nm wavelengths using a UV-Vis (Shimadzu Uvmini-1240) spectrophotometer.

Fourier-transform infrared spectroscopy was employed to perform the chemical analysis of the functional groups present in the materials used and the FeNPs obtained in the project. Powder samples (FeCl_3_·6H_2_O and Fe_2_O_3_ nanoparticles) were analyzed using ATR (attenuated total reflectance). The analysis used an IR-Prestige 21 FTIR Spectrophotometer (Shimadzu, Kyoto, Japan).

Scanning electron microscopy was utilized to characterize the surface morphology, phase purity, and particle size of the FeNPs using a Thermo Fisher Scientific (Waltham, MA, USA) (FEI) Quattro S SEM.

Particle size analysis was carried out to determine the particle size of the synthesized nanoparticles using a zetasizer, a Malvern Instruments device (Worcestershire, UK).

A physical property measurement system (Quantum Design PPMS 6000) from Quantum Design (San Diego, CA, USA) was used to measure the synthesized material’s magnetic, thermal, and electrical transport properties via AC/DC magnetometry under controlled magnetic field and temperature conditions. Magnetization properties such as saturation magnetization (Ms), coercive field (Hc), and remanence magnetization (Mr) were performed using the physical property measurement system (Quantum Design PPMS 6000) from Quantum Design at 300 K.

Electron spin resonance was applied to observe the processes occurring when electromagnetic waves in the microwave region were absorbed by electron spins in a magnetic field. The ESR measurements were performed using a JEOL (Brand-JES-FA300, Tokyo, Japan) electron spin resonance spectrometer with a Bruker EMX (X-band Spectrometer, Billerica, MA, USA). The ESR spectra were recorded at room temperature (296 K) and in the range of 0–1000 mT of DC magnetic field. All the samples were loaded into quartz ESR tubes, and the magnetic contributions of the cavity and the quartz tubes were extracted from measurements.

Thermogravimetric Analysis was conducted to determine the mass losses or gains in materials as a function of temperature or time. The thermal behavior of the FeNPs was identified with a thermogravimetric analyzer (Seiko Instruments SII Exstar 6300 TG/DTA, Chiba, Japan) system.

X-ray Diffraction Analysis (Malvern PANalytical X’Pert PRO, Worcestershire, UK) was used to analyze the samples’ crystalline phases, as each crystalline phase reflects X-rays in a characteristic pattern depending on its atomic arrangement.

### 2.4. Assessment of Cytotoxicity and Anticancer Potential of Green-Synthesized FeNPs

Cytotoxicity assays of the extracts and FeNPs produced from these extracts were performed using C6 and L929 cell lines. The 3-(4,5-dimethylthiazol-2-yl)-2,5-diphenyltetrazolium bromide (MTT) method, first described by Mosmann [21] and later developed by Alley and colleagues, is an indirect method used to evaluate cell growth and/or cell death [22]. C6 and L929 cells grew in DMEM-F12 medium containing 5% FCS. After 4–6 days, the cells were taken using trypsin-EDTA and seeded into 96-well plates at a density of 10^4^ cells per well. The cells were incubated for 24 h at 37 °C in 5% CO_2_ conditions. After incubation, the characterized FeNPs were prepared, sterilized for cytotoxicity analysis, and added to the wells in concentrations ranging from 1 to 100 µg/mL. Each concentration was tested in quadruplicate. Following 24 and 48 h incubations, 10 µL of MTT (10 mg/µL) stock solution was added to each well, and the plates were incubated in the dark for 3–4 h. Afterward, 100 µL of DMSO was added to dissolve the formazan crystals formed on the cells. The plates were kept at room temperature in the dark for 30 min. The absorbance was measured at 540 nm using an ELISA microplate reader (Thermo Scientific Multiskan, Waltham, MA, USA), and the results were compared with control groups that did not receive any treatment to calculate the percentage of cell viability.

## 3. Results and Discussions

### 3.1. UV-Vis Spectrophotometry Analysis

**Figure 1:**
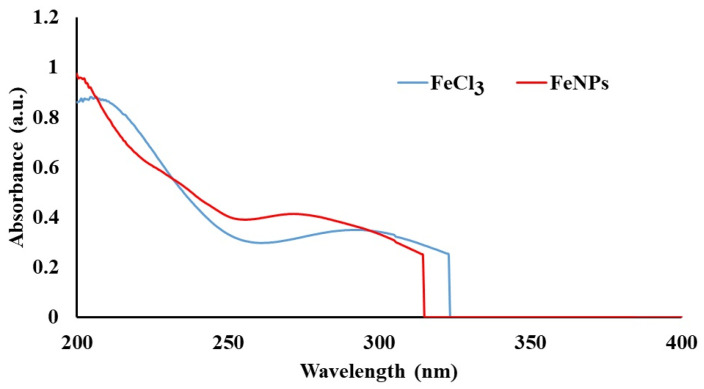
UV-Vis absorbance spectra of FeCl_3_ and FeNPs synthesized using extracts of *Vitex agnus-castus* seeds.

The UV-Vis absorption spectra of the FeCl_3_ solution and FeNP suspensions synthesized using *Vitex agnus-castus* seed extracts are shown in Figure 1. The spectra of the ferric chloride and FeNP solutions were recorded using UV-Vis spectrophotometry in the 200–800 nm wavelength range. The *Vitex agnus-castus* seed extracts exhibited peaks at 263 and 315 nm, corresponding to various phyto-moieties in the extract. These peaks disappeared after the addition of the FeCl_3_ solution, indicating interactions between the metal ions and the functional groups of the phyto-moieties in the plant extract. This interaction facilitated the reduction process and the formation of FeNPs, with the plant extract acting as a capping agent to prevent agglomeration. A peak corresponding to ferric chloride was observed at 296 nm, while a peak characteristic of iron oxide was detected at 272 nm [23]. The shift in wavelength and the changes in peak characteristics indicated the successful formation of nanoparticles.

An abrupt drop in UV absorbance is observed around the 300 nm region in Figure 1 for both the FeNP and FeCl_3_ solutions. This behavior is likely related to the limitations of the UV-Vis spectrophotometer used in this study, where a transition between UV and visible light detectors can cause noise or inconsistencies in the absorbance spectrum. Additionally, this drop may reflect the intrinsic optical properties of the samples. In FeNPs, the stabilization by plant-derived molecules could result in reduced plasmon resonance beyond specific wavelengths, leading to a sharp cutoff. Similarly, the FeCl_3_ solution may exhibit weak absorbance in this region, contributing to the observed pattern. Importantly, this phenomenon does not interfere with the key spectral features relevant to FeNP synthesis, such as characteristic absorbance shifts, and the data remain valid for interpretation. Similar effects of concentration and environmental changes on UV-Vis absorbance have been reported by Tong et al. [24], and abrupt changes related to nanoparticle properties have been observed in studies by Bratovcic [25].

### 3.2. Fourier-Transform Infrared Spectroscopy Analysis

**Figure 2:**
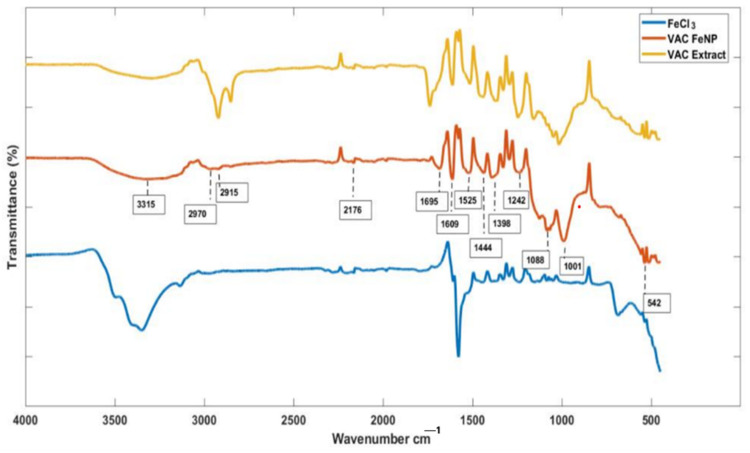
The FTIR patterns of FeNPs.

Fourier-transform infrared (FT-IR) spectrometry was conducted to identify the functional groups of the phyto-moieties in the *Vitex agnus-castus* seed extracts responsible for reducing metal ions. Figure 2 shows the FTIR spectra of the *Vitex agnus-castus* extract and the synthesized FeNPs. Asymmetric and symmetric N-O stretching was identified at 1550 cm^−1^ and 1350 cm^−1^, while N-H and O-H stretching were observed in the 3000–3500 cm^−1^ range, suggesting the presence of phenolic and amine groups, which play a critical role in the reduction of Fe^3+^ ions and the stabilization of nanoparticles. Additionally, C≡N stretching in the functional groups was noted within the broad band of 2200–2300 cm^−1^. In line with these spectra, it is suggested that similar peaks in both the *Vitex agnus-castus* extract and the FeNP spectra arise from the adsorption of plant-derived structures onto the nanoparticle surface [26]. Based on the bands, it was confirmed that there are functional groups in the structure of the *Vitex agnus-castus* extract, including aromatics, phenols, amines, hydroxyl groups, carboxyl groups, carbonyl groups, and aliphatic hydrocarbon groups [27]. These functional groups likely act as reducing agents and capping agents, ensuring the stability and functionality of the FeNPs. On the other hand, in the FTIR spectra of the FeNPs, characteristic peaks corresponding to alkanes (1340–1470 cm^−1^, 2850–2970 cm^−1^), alkynes (2100–2260 cm^−1^), carbonyl groups (1690–1760 cm^−1^), C=C alkenes (1610–1680 cm^−1^), and C=C aromatic rings (1500–1600 cm^−1^) were observed (Table 1). These peaks suggest the successful incorporation of organic molecules from the plant extract onto the nanoparticle surface. Furthermore, peaks associated with aromatic amines (1300–1370 cm^−1^), amine–amide groups (1180–1360 cm^−1^), alcohols, carboxylic acids (1050–1300 cm^−1^), and aliphatic amines (1040–1053 cm^−1^) highlight the functionalization of the FeNP surface, contributing to their stability and potential biomedical applications [28]. The most prominent peaks in the FTIR spectra of the FeNPs belong to the metal–oxygen (Fe-O) vibration modes, confirming the successful formation of iron nanoparticles. These peaks generally occur between 400 cm^−1^ and 600 cm^−1^, with the specific peak observed at approximately 542 cm^−1^ in this study. This observation validates the synthesis process and supports the hypothesis that plant-derived functional groups interact with the nanoparticle surface, ensuring their stability and functional integrity [29].

### 3.3. Scanning Electron Microscopy Analysis

The morphology and size distribution of the FeNPs were examined using scanning electron microscopy and zetasizer analysis. The magnetic dipole interactions on the nanoparticles, particularly the formation of ring-shaped structures, were investigated. The SEM images and zeta results provided comprehensive information on the nanoparticles’ size distribution and surface morphology. A previous TEM study demonstrated that magnetic nanoparticles tend to self-assemble into rings, chains, and other two-dimensional structures. It was suggested that magnetic dipole interactions are responsible for these unique arrangements of nanoparticles in ring and chain formations [30].

SEM images are instrumental in observing the morphological characteristics of particles. The ring-shaped structures could result from a specific growth mechanism or spontaneous organization of the nanoparticles. This structure may indicate that the particles are organized on the surface with a certain geometry or exhibit a regular morphology influenced by environmental factors [31]. From the SEM micrographs, the synthesized nanoparticles exhibit a round and cavity-like morphology with varying sizes compared to those produced through chemical synthesis. The size variation arises from the limited capacity of secondary metabolites to regulate particle dimensions in phyto-mediated synthesis, which, despite being a green method, differs from chemical synthesis in this regard. The plant extract, rich in diverse secondary metabolites, serves as a both reducing and stabilizing agent during the bio-reduction process involved in the formation of metallic nanoparticles [32].

A likely explanation is that the diverse polyphenols present in the extracts have the potential to significantly influence the shape and size of iron oxide nanoparticles. The lower capping ability of the plant extract and agglomeration tendency of the FeNPs due to magnetic dipolar interactions can also take a role in the formation of particles [33,34]. The average particle size of 398.9 nm observed in the zeta analysis and the ring-shaped structures seen in the SEM images indicate that the particles formed large clusters organized into regular geometries on the surface. This observation aligns with the ring-shaped structures noted in the SEM images. A Polydispersity Index (PdI) value of 0.337 suggested that the particle sizes were not uniform, meaning that both large and small particles were observed together in the SEM images. The data obtained from the zeta analysis were consistent with the SEM images. The ring-shaped nanoparticles may indicate that the growth conditions allowed the particles to form regular geometric structures, suggesting a tendency for the FeNPs to self-organize on the surface. This was most likely because the *Vitex agnus-castus* extract was a combination of various naturally occurring compounds with different reducing properties. The elements shown in Table 2, such as Mg, K, and Ca, are believed to originate from the *Vitex agnus-castus* extract, as it naturally contains minerals and bioactive compounds absorbed from its environment. These plant-derived elements likely contributed to the reduction and stabilization processes during the synthesis of the nanoparticles. The polyphenols or antioxidants present in the extract were crucial in preventing the agglomeration of nanoparticles, enhancing their dispersion by functioning as capping agents.

**Figure 3:**
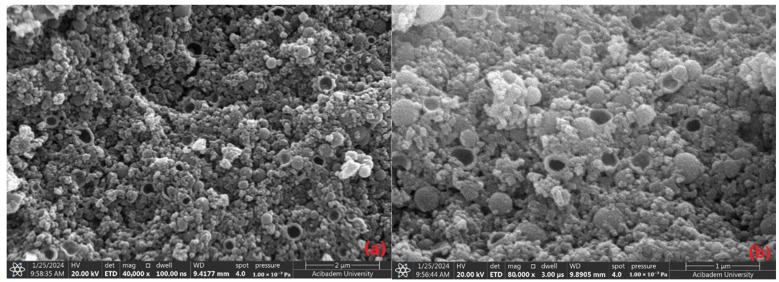
SEM images of FeNPs at different magnifications: 40.000× (**a**) and 80.000× (**b**).

In conclusion, the SEM images in Figure 3 detail the surface morphology and organization of the nanoparticles. At the same time, the zeta analysis provided insights into the particle size distribution and their colloidal stability in solution. The average particle size obtained in the measurements made with the zetasizer was large due to the agglomeration of nanoparticles (coming together to form larger structures). Magnetic nanoparticles tend to agglomerate spontaneously in solution due to magnetic dipole interactions. This leads to larger hydrodynamic diameters in DLS measurements. The examination of the SEM images revealed that the nanoparticles were actually much smaller in size than suggested by the zetasizer. The images also showed that the iron nanoparticles spontaneously came together to form ‘ring-shaped’ structures. This formation is attributed to the arrangement of nanoparticles as a result of magnetic dipole interactions, and similar observations have been reported in the literature. As a result, it is thought that the large size values obtained with the zetasizer are due to the agglomeration effect, and the real nanoparticle sizes are more accurately represented by SEM analyses [35]. When these results are evaluated together, it can be concluded that the FeNPs exhibited a specific arrangement, although their sizes were not homogeneous.

The dispersion and aggregation of magnetic nanoparticles introduce variability in their measured magnetic properties, as the nanoparticles differ in size, shape, and interactions, leading to non-uniform behavior [36]. Notably, any alteration in the size or shape of a nanoparticle significantly impacts its inter-particle interactions and absorption characteristics. To enhance practicality, nanoparticles must undergo surface functionalization to minimize agglomeration, improve biocompatibility, prevent protein adsorption, reduce toxicity, and extend their circulation time in the bloodstream [37]. In the absence of surface modifications, magnetic nanoparticles with hydrophobic surfaces and high surface-to-volume ratios tend to aggregate, forming large clusters [38]. For instance, in clinical applications, superparamagnetic iron nanoparticles (FeNPs) are widely utilized in hyperthermia treatments due to their high magnetic saturation. Extensive research has focused on optimizing the specific absorption rate (SAR) by synthesizing nanomaterials with precise shapes, sizes, and surface modifications, as the size-dependent properties of magnetic nanoparticles play a critical role in localized cell heating [39].

Parameters such as the selection of plant parts, using appropriate solvents for the extraction process, filtration/chromatography to eliminate any impurities, metal salt solution of choice as the nanoparticle precursor, appropriate temperature, and pH of the reaction can be improved for the formation of highly uniform and dispersion-sized NPs.

### 3.4. Particle Size (Zetasizer) Analysis

The data in Table 3 show the size and PDI values of the nanoparticles synthesized using the green synthesis method without any stabilizer or surfactant. The Z-Ave values ranged from 390.4 nm to 442.3 nm, indicating that the nanoparticles were synthesized homogeneously, with sizes within quite a close range. The PDI values ranged from 0.331 to 0.427, suggesting a slight heterogeneity in size distribution [40,41]. Despite the absence of a stabilizer in the green synthesis method, the similar sizes of the nanoparticles suggest that the natural stabilization effect of the biomolecules might have had a minimal influence. However, synthesis conditions could be adjusted to reduce the particle size further and achieve a more homogeneous size distribution. For example, lower synthesis temperatures or shorter reaction times may lead to the formation of smaller nanoparticles.

Optimizing the pH of the reaction medium could limit particle growth and help achieve smaller sizes. Additionally, using polymer- or biopolymer-based stabilizers to control size could prevent particle agglomeration and contribute to producing smaller and more homogeneous nanoparticles. Such optimizations would allow nanoparticle sizes and properties to be tailored to specific application areas.

**Figure 4:**
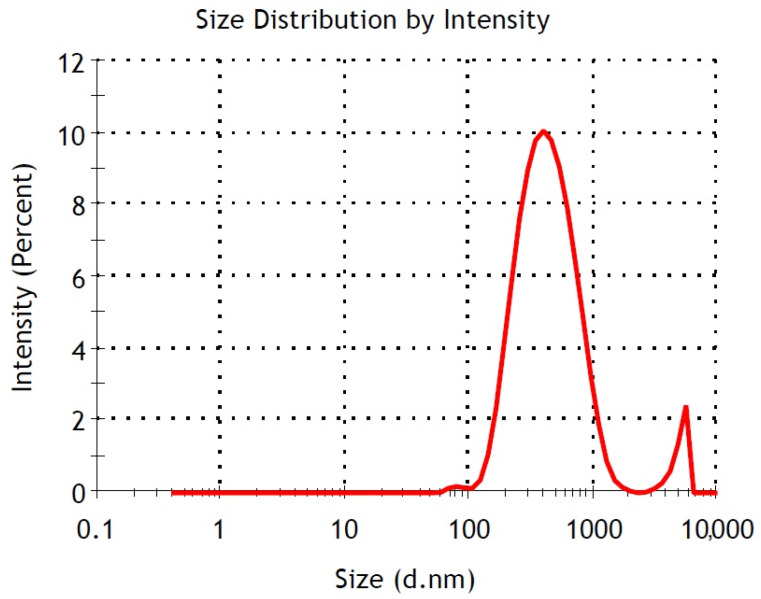
Particle size analysis of FeNPs.

The size analysis shown in Figure 4 corresponds to FeNPs with a size of 398.9 nm. This value indicates that the FeNPs are relatively larger than suggested by the SEM analyses. It was considered that the nanoparticles might form larger macroscopic clusters or have a tendency to aggregate. The Polydispersity Index (PdI) of 0.337 indicates a moderate size distribution among the nanoparticles. While a PdI value below 0.1 indicates a uniform system, values above 0.4 suggest a heterogeneous distribution. A PdI value of 0.337 implies that the particle sizes exhibit some variation, meaning that certain particles are either larger or smaller.

The zeta potential of the synthesized iron nanoparticles was measured as −10.5 mV, which indicates borderline electrostatic stability. In colloidal systems, zeta potential values between −10 mV and −20 mV generally mean that the repulsive forces between particles are weak, making it easier for them to clump together [42]. This explains the large hydrodynamic size observed in the DLS measurements, likely caused by the nanoparticles clustering due to magnetic dipole interactions. The borderline stability suggested by this zeta potential could also be influenced by factors like the ionic strength of the solution, its pH, or the lack of stabilizing agents or surface modifications. These conditions seem to encourage the observed agglomeration, as magnetic nanoparticles naturally tend to form clusters. The SEM analysis supports this, revealing smaller individual particles and showing evidence of these particles coming together to create ring-shaped structures. This behavior matches what has been reported in the literature about the self-assembly tendencies of magnetic nanoparticles. In conclusion, the measured zeta potential suggests that while the suspension may remain stable for a short time, achieving long-term stability would require surface modifications or adjustments to the solution, such as optimizing the pH or adding dispersing agents.

### 3.5. Physical Property Measurement System Analysis

**Figure 5:**
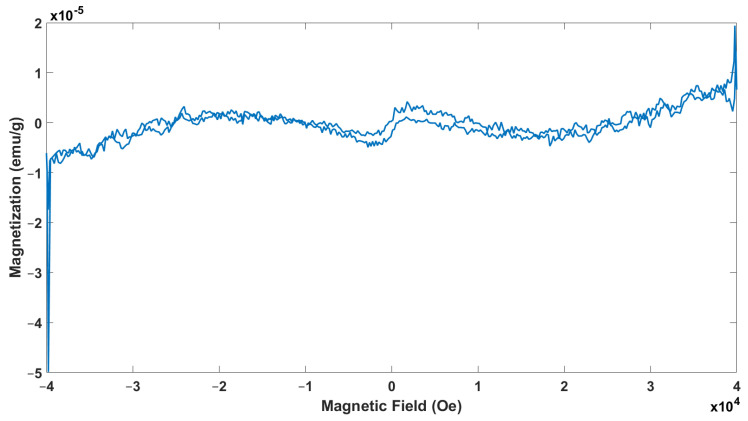
Magnetization measurement of FeNPs.

Magnetization measurements were performed using the PPMS to gain deeper insights into the magnetic properties of the synthesized nanoparticles. The room-temperature PPMS analysis of the FeNPs is shown in Figure 5. A strong external magnetic field of +1 Tesla was applied to the system, and once positive saturation was reached, the longitudinal hysteresis loop was recorded. The magnetization curve displays a narrow hysteresis loop under the applied magnetic field, indicating that the sample exhibits soft ferromagnetic behavior at room temperature, characterized by Mr and Hc, as shown in the accompanying figure. The green synthesis of the nanoparticles did not transform the Ms value significantly. The magnetic properties of ferrite materials are typically influenced by various factors, including the synthesis method, crystallite size, sintering temperature, cation redistribution among occupancy sites, nanoparticle morphology, and interactions between nanoparticles. These interactions are often associated with surface atoms, as well as dipolar and exchange coupling effects. A reduction in saturation magnetization in nanoparticles is commonly linked to surface effects and the spin configuration of the nanoparticles [43]. The hysteresis loops (300 K) highlight the soft ferromagnetic behavior of this precursor that contains a large amount of organic ligand and the presence of a small amount of impurities which are not superparamagnetically relaxed, even at this temperature. In the present samples, the lower values of *Ms* of these samples can also be accredited to the sub-lattice exchange, the interaction amongst the cations, and the replacement of non-magnetic ions with ferrite ones. The decrease in Ms results from the organic substances corresponding to the sample’s phase and morphology. This soft ferromagnetism can be attributed to the nanoscale materials’ high surface-to-core atom ratio standard. In particular, the 4d and 5d transition metals, characterized by reduced coordination geometry, exhibit surface-induced spin polarization, potentially leading to nanoparticle ferromagnetic ordering [44]. The lack of clear saturation magnetization and coercivity values in this study might be explained by the specific synthesis conditions and the size of the particles, which could affect how well the magnetic order forms. The balance between surface effects and core properties in nanoparticle systems often determines the overall magnetic behavior. With their unpaired spins and reduced coordination, surface atoms tend to dominate magnetic responses, particularly in small particles. As a result, the weak ferromagnetism observed in this case could also be due to particle agglomeration, spin canting, or surface oxidation—common effects seen in FeNPs [18].

Further studies that include temperature-dependent magnetization and coercivity measurements, as well as high-resolution structural analysis, would be highly beneficial to fully understand the magnetic properties of these nanoparticles and their potential applications in magnetic hyperthermia or magnetic resonance imaging (MRI) [45].

### 3.6. Electron Spin Resonance Spectroscopy Analysis

**Figure 6:**
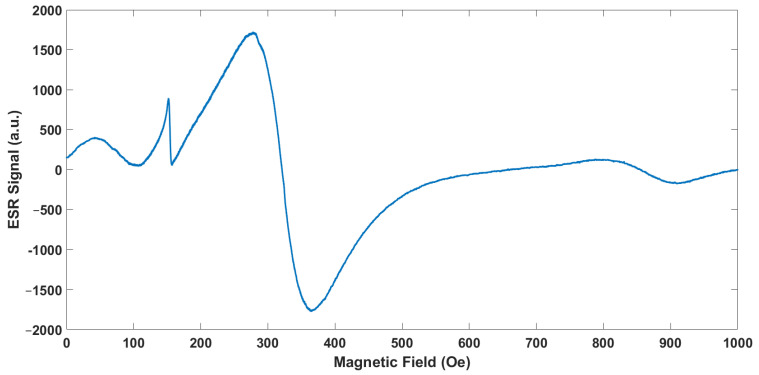
ESR spectra of FeNPs.

To obtain information about the spin dynamics of nanosized systems, the ESR spectra were measured at room temperature at 300 K with a frequency of 9.11 GHz. The ESR spectra of the sample, as illustrated in Figure 6, showed a pattern with multiple peaks, typical of systems containing unpaired electrons in diverse magnetic environments. The magnetic field strength was plotted on the x-axis, while the y-axis displayed the signal intensity, revealing how the sample absorbed microwave radiation as the magnetic field changed. Smaller, sharp peaks were observed in the low-field range (100–250 G), suggesting localized interactions or paramagnetic centers with weak magnetic coupling. These peaks were likely due to surface-bound species or impurities that added to the overall signal without causing broadening. Such low-field sharp signals were commonly linked to unpaired electrons in isolated paramagnetic sites, indicating specific localized magnetic interactions [46].

The central region of the ESR spectra (300–400 G) showed the most significant signal, marked by a clear positive-to-negative transition. This peak highlighted the primary resonance condition for the paramagnetic species in the sample. The intensity value of the peak for the sample is about 1700 au. The intensity and sharpness of this feature suggested a large number of unpaired electrons situated in a fairly uniform magnetic environment. Such central resonance peaks are typically observed when unpaired electrons experience minimal anisotropy, resulting in distinct and clear signals [47]. A broader and less intense peak appeared in the high-field range (500–800 G). This broadening likely stemmed from stronger dipolar interactions between the paramagnetic centers or magnetic anisotropy affecting the unpaired electrons. Broad peaks like these are commonly found in systems where the magnetic environment is more varied, potentially due to interactions among neighboring paramagnetic sites or structural differences within the material. These observations align with findings from other studies showing that inter-particle magnetic interactions or dipole–dipole coupling significantly impact the magnetic behavior in such systems [46]. Overall, the ESR analysis painted a comprehensive picture of the magnetic properties of the nanoparticles. It indicated the presence of both isolated paramagnetic centers and more complex interactions resulting from dipolar coupling or structural variation. The combination of sharp and broad signals across different magnetic field regions supported the idea of a heterogeneous magnetic environment within the sample [48].

### 3.7. Thermogravimetric Analysis

**Figure 7:**
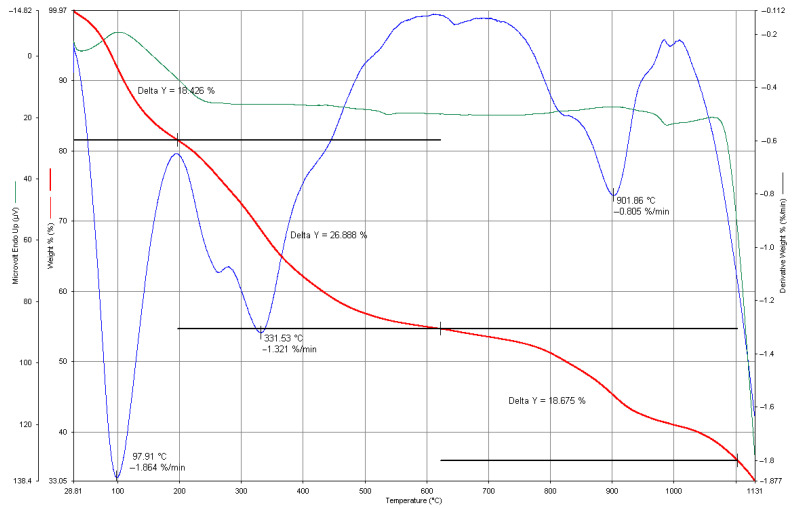
Thermogravimetric analysis curve of FeNP sample.

The graph in Figure 7 presents the TGA data of the FeNPs, displaying three distinct sets of data: The red curve represents the change in the sample’s weight percentage (weight %) with increasing temperature. This curve indicates mass loss as the temperature rises. According to the graph, three distinct mass loss regions were identified: a 1.864% mass loss at 97.91 °C, which likely corresponded to the evaporation of adsorbed water or other low-molecular-weight components; a 1.321% mass loss at 331.53 °C, potentially due to the decomposition of organic compounds or the detachment of stabilizing agents on the nanoparticle surfaces; and a 0.805% mass loss at 901.86 °C, possibly attributed to the breakdown of more thermally stable compounds. The blue curve corresponds to the derivative of the weight loss (DTG, Derivative Weight %), showing the mass loss rate at various temperature intervals. The data indicate that the most rapid mass loss occurred at 97.91 °C at −1.864%/min, with additional peaks in the rate observed near 331.53 °C and 901.86 °C. The green curve recorded a microvolt parameter (μV), likely reflecting the sample’s thermal signal or response as a temperature function.

The results of the TGA analysis revealed that the FeNPs experienced weight loss at varying temperature levels. The initial decrease in weight could be attributed to the evaporation of water or organic elements. As the temperature increased, weight loss occurred due to the breakdown of these substances. The last phases of weight loss hinted at the breakdown of compounds. This implies that there might have been molecules on the nanoparticle surfaces that could be compounded, prompting this decomposition pattern [13].

During the synthesis process, plant extracts, polyphenols, and various organic compounds present in the plants can adhere to the surfaces of nanoparticles, leading to a decrease in mass, as seen in the Thermogravimetric Analysis (TGA). Components like terpenoids, flavonoids, proteins, and other organic substances typically found in plant extracts are known to break down within the temperature range of 200 °C to 500 °C, causing a mass loss within this interval [49]. Specifically, polyphenols and flavonoids, which help stabilize the nanoparticles during synthesis, often decompose at lower temperatures, leading to an observable mass loss in the analysis [50]. Plant-based compounds like tannins, alkaloids, and terpenoids play a crucial role in synthesizing nanoparticles, acting as surface stabilizers [51]. The mass loss observed at lower temperatures in the TGA analysis is likely due to the decomposition of these organic compounds. Polyphenols and similar molecules are not heat-resistant and break down within specific temperature ranges. As temperatures increase, the mass loss might be linked to the breakdown of more robust parts of the nanoparticles, such as their metallic components. This suggests that plant-derived compounds on the surface could explain some of the mass loss seen in the TGA results. Aside from polyphenols and flavonoids, other bioactive compounds from plant extracts may also be present on the nanoparticle surfaces, contributing to mass loss. These include organic molecules like tannins, alkaloids, saponins, terpenoids and proteins. For instance, terpenoids and proteins often decompose at higher temperatures, adding to the mass loss seen in the TGA analysis. Saponins, which act as surface-active agents, can attach to the nanoparticles and influence their stability, resulting in the varied thermal behavior in the analysis [24,52]. The presence of these organic compounds might explain the gradual mass losses recorded in the TGA. Specifically, terpenoids commonly found in plant extracts begin to decompose at higher temperatures, typically within the 200 °C to 400 °C range [53]. Proteins from plant sources also aid in stabilizing nanoparticles and generally decompose between 200 °C and 500 °C, contributing to the observed mass loss [54].

### 3.8. X-Ray Diffraction Analysis

**Figure 8:**
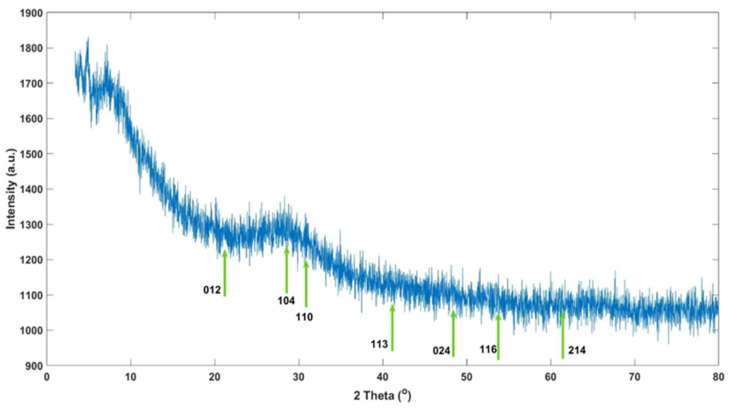
XRD spectra of FeNPs.

The XRD analysis of the synthesized iron oxide nanoparticles revealed peaks typically associated with well-known iron oxide phases such as hematite (α-Fe_2_O_3_) and maghemite (γ-Fe_2_O_3_). These nanoparticles, synthesized via green synthesis methods, displayed characteristic XRD patterns corresponding to these phases, consistent with previously reported findings. For the hematite (α-Fe_2_O_3_) phase, the characteristic XRD peaks were observed at various planes: the (012) plane at 24.1°, (104) plane at 33.1°, (110) plane at 35.6°, (113) plane at 40.8°, (116) plane at 54.1°, and (214) plane at 62.4° [55]. Similarly, for the maghemite (γ-Fe_2_O_3_) phase, the following characteristic XRD peaks were noted: the (220) plane at 30.2°, (311) plane at 35.6°, (400) plane at 43.2°, (422) plane at 53.6°, (511) plane at 57.3°, and (440) plane at 62.9° [56]. These results suggest that both hematite and maghemite phases are present in the nanoparticles, as indicated by the alignment of the observed XRD peaks with these characteristic planes. This aligns with common findings in synthesizing iron oxide nanoparticles through green chemistry methods. In the XRD results depicted in Figure 8, the observed peaks are compared with the characteristic peaks reported in the literature for the hematite (α-Fe_2_O_3_) and maghemite (γ-Fe_2_O_3_) phases to characterize the phase structure of the synthesized iron oxide nanoparticles. The peaks observed at 22.76° and 23.11° are close to the (012) plane of the hematite phase (24.1°), suggesting the formation of the hematite phase in the synthesized nanoparticles. The peak at 29.73° corresponds to the (220) plane of the maghemite phase (30.2°), confirming the structural presence of the maghemite phase in the nanoparticles. The peak at 40.92° strongly correlates with the (113) plane of hematite (40.8°), serving as another indicator of the hematite phase.

Additionally, the peak at 42.07° is compatible with the (400) plane of maghemite (43.2°). The peaks at 58.42° and 60.03° align with the (511) and (440) planes of maghemite (57.3° and 62.9°, respectively), further suggesting the dominance of the maghemite phase in the synthesized nanoparticles. The 65.06° and 79.27° peaks do not match the characteristic peaks of the hematite or maghemite phases and may indicate secondary phases or impurities. The XRD results revealed the presence of both hematite (α-Fe_2_O_3_) and maghemite (γ-Fe_2_O_3_) phases in the synthesized iron oxide nanoparticles. Notably, the peaks observed between 22.76° and 42.07° reflect the structural characteristics of these two iron oxide phases. The peaks observed at higher angles may point to secondary phases or impurities that arose during the synthesis process. These findings support that the synthesis method produces delicate crystal structures containing hematite and maghemite phases. The crystallite sizes specific to iron oxide nanoparticles were calculated using the Debye–Scherrer formula from the full width at half maximum (FWHM). Upon evaluation, the crystallite sizes derived from different 2θ angles varied significantly. Specifically, the crystallite size at 42.07° was approximately 104 nm, while at 65.06°, it increased to 921 nm. These observations indicate that the crystallite sizes vary depending on the angular positioning of the peaks, suggesting a heterogeneous size distribution of the synthesized nanoparticles.

In the green synthesis of iron oxide nanoparticles, organic materials from plant extracts play a role in their formation. However, these organic compounds can also lead to the development of other phases or amorphous impurities alongside the pure iron oxide structure during the synthesis process [55]. Such compounds, like flavonoids and polyphenols in the extract, might interact with the nanoparticles, resulting in secondary phases [57]. In XRD analysis, this interaction may show up as broad peaks, indicating the presence of amorphous structures or organic components mixed with the main hematite (Fe_2_O_3_) or maghemite (Fe_3_O_4_) phases [58]. These peaks typically have low intensity and appear at lower angles [59]. Iron oxide nanoparticles synthesized via green synthesis methods typically exhibit small crystallite sizes [60]. These small sizes manifest as broader and less sharp peaks in XRD analysis [56]. While the XRD peaks of typical iron oxide phases such as hematite or maghemite are present, the broadness of these peaks suggests small crystallite sizes in the nanoparticles [50]. The sizes calculated using the Scherrer formula indicate small crystallite dimensions [61].

In addition to the peaks of different iron oxide phases (hematite (α-Fe_2_O_3_) and maghemite (γ-Fe_2_O_3_)), broad and low-intensity peaks may be observed due to amorphous carbon structures or other organic materials originating from the plant extract [55,56]. This suggests that secondary phases and delicate crystalline structures are also present alongside the synthesized iron oxide nanoparticles. This indicates that in addition to the iron oxide nanoparticles produced by green synthesis, secondary phases from the plant extract and small crystallite sizes are evident [62]. The XRD results suggest the presence of organic materials or impurities in addition to these small-sized iron oxide nanoparticles [63]. This suggests that organic coatings or stabilizing structures may be present around the nanoparticles [50]. Based on the XRD results, the synthesized iron oxide nanoparticles reflect both iron oxide and secondary phases [57]. Due to the use of green synthesis methods, the organic components in the plant extract may have promoted the formation of secondary phases; this is evident in the observation of various peaks and broad crystalline structures in the XRD analysis [60].

### 3.9. Assessment of Cytotoxicity and Anticancer Activity in Cells

**Figure 9:**
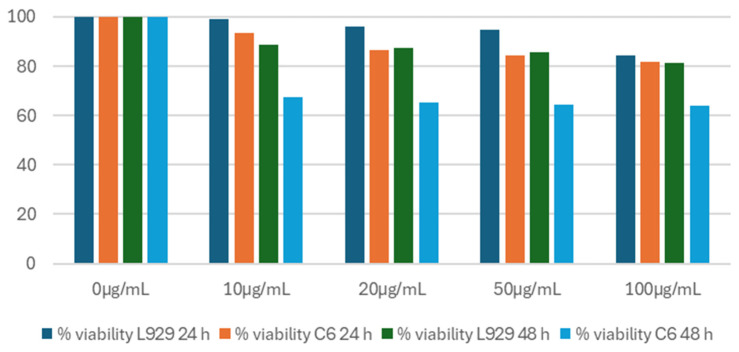
Percentage viability of L929 and C6 cells at 24 and 48 h.

MTT assays were applied in vitro to assess the cytotoxic effects of the FeNPs on (L929) mouse fibroblast and (C6) glioma cells. In this context, IC_50_ (50% inhibitory concentration) values were determined at the 24th and 48th hours after the treatment of NPs with various concentrations. The graph in Figure 9 presents the percentage viability of the L929 (dark blue bar @24 h and green bar @48 h) and C6 cells (orange bar @24 h and light blue bar @48) at 0–100 µg/mL concentrations. Based on the viability data, the IC50 evaluations indicated that for L929 cells, the IC50 value was 26.51 µg/mL at 24 h and 12.46 µg/mL at 48 h.

For the glioma C6 cells, the IC50 values were 10.73 µg/mL at 24 h and 5.92 µg/mL at 48 h. Upon evaluating the results, it was observed that the FeNPs were more than twice as effective on the cancer cell line C6 glioma cells compared to the healthy fibroblast L929 cells at 24 h. Furthermore, at 48 h, the effectiveness on the C6 glioma cells was nearly double that of the L929 cells. Thus, in vitro evaluations at both 24 and 48 h demonstrated that FeNPs are biocompatible and have cytotoxic effects on cancer cells. Additionally, the increased efficacy of the FeNPs at 48 h for both cell lines was observed.

This section details how FeNPs exhibit cytotoxic effects on cancer cells and biocompatibility, making them a promising candidate for anticancer applications. However, further studies are needed to elucidate the underlying mechanisms and optimize the design of green-synthesized FeNPs for cancer therapy applications.

Physicochemical parameters such as size, shape, and surface properties play a critical role in understanding the biological effects of nanoparticles. In particular, the effects of nanoparticle size on biological activity have been associated with an increase in surface area [64]. Some studies in the literature aim to gain insights into the biological effects of nanomaterial size. A study on titanium dioxide (TiO_2_) and aluminum oxide (Al_2_O_3_) nanoparticles reported that for this type of nanomaterial, biological activity increases as particle size decreases. For these nanoparticles, it was observed that 5 nm TiO_2_ particles caused a greater reduction in cell colony number compared to 200 nm particles, and a similar trend was observed for 10 nm Al_2_O_3_ particles. This finding suggests that smaller particles result in higher toxicity due to increased interactions with biological systems [65]. Similarly, research on silver nanoparticles demonstrated that smaller particles exhibit faster ion release, leading to higher biological activity. For instance, among spherical silver nanoparticles of 7 nm, 29 nm, and 89 nm sizes, 7 nm particles were reported to have stronger toxicity and antibacterial effects. Additionally, a comparative study on zinc oxide (ZnO) and silica (SiO_2_) particles revealed that nano-sized particles (ZnO: 100 nm, SiO_2_: 10–20 nm) displayed higher toxicity than micron-sized particles (ZnO: 5 μm, SiO_2_: 10 μm). This effect was validated through experiments on human lung epithelial cells (L-132) and human monocytes (THP-1), indicating that nano-sized particles are more toxic than their micron-sized counterparts [66]. Not only size, but also shape and surface properties significantly impact the biological effects of nanoparticles. For example, differences in ion release rates based on the shape of silver nanoparticles have been reported to influence toxicity levels. The toxicity effects of spherical, rod-shaped, and triangular silver nanoparticles were linked to surface atom density [67]. It has been shown that nanomaterials cause higher toxicity compared to micron-scale particles. A study on ZnO and SiO_2_ particles confirmed that nano-sized particles exhibit greater toxicity than micron-sized ones, with this effect being concentration-dependent [66]. These findings also highlight variations in cellular sensitivity to particles of the same composition. Understanding the toxicity of nanomaterials is critically important for safety evaluations in environmental and biomedical applications [68]. It is evident that in addition to size, the effects of shape, surface properties, and cell type need to be more comprehensively studied in future research.

## 4. Conclusions

This study showed that FeNPs can be successfully produced using the green synthesis method using *Vitex agnus-castus* plant extract. The synthesized nanoparticles were examined in terms of biocompatibility and magnetic properties, and their potential for biomedical applications was shown. The results obtained by characterization techniques such as UV-Vis spectroscopy, FTIR, and SEM showed that the nanoparticles had a moderately homogeneous distribution and that the FeNPs exhibited the expected structural and magnetic properties. In particular, the SEM results revealed the surface morphology and shell structure of the nanoparticles, while zetasizer analyses confirmed the colloidal stability and distribution of the particles.

*Vitex agnus-castus* plant extract serves as a bio-reductant and coating agent in the synthesis of nanoparticles, resulting in the reduced agglomeration of nanoparticles. This coating was also supported by TGA results showing the presence of organic molecules on the surface of the nanoparticles. In this context, herbal compounds play a critical role in increasing the colloidal stability and biocompatibility of synthesized nanoparticles. The magnetic properties of FeNPs are of great importance for use in biomedical applications, such as hyperthermia and magnetic tracer agents in MRI and MPI systems. Their low toxicity and high biocompatibility indicate that these nanoparticles could be a potential tool in diagnosing and treating cancer.

## Figures and Tables

**Table 1:** FTIR spectral bands and functional groups observed in synthesized FeNPs [26].

Wavenumber (cm^−1^)	Functional Group	Role
3000–3500	O-H and N-H	Reducing agent, stabilizer
1690–1760	C=O (Carbonyl)	Interaction with nanoparticle surface
1500–1600	Aromatic C=C	Adsorption onto surface, stabilizer
1340–1470, 2850–2970	Alkanes	Stabilizing coating
2200–2300	C≡N	Presence of organic functional groups
1040–1300	Carboxylic acids, alcohols	Binding to nanoparticle surface
400–600	Fe-O	Chemical signature of nanoparticle formation

**Table 2:** Elemental composition of synthesized nanoparticles using *Vitex agnus-castus* extract (SEM-EDX analysis).

Element	Weight %	Atomic %
Mg	23.1	37.1
K	16.6	16.5
Ca	16.4	16.0
Fe	43.8	30.4

**Table 3:** Size and pDI values of synthesized nanoparticles.

FeNPs	1	2	3	4	5	6	7	8
Z-Ave (nm)	442.3	398.9	390.5	390.4	393.3	421.4	421.8	428.5
pDI	0.360	0.337	0.342	0.351	0.343	0.331	0.427	0.373

## Data Availability

The original contributions presented in the study are included in the article, and further inquiries can be directed to the corresponding author.
